# Electroacupuncture treatment ameliorates depressive-like behavior and cognitive dysfunction via CB1R dependent mitochondria biogenesis after experimental global cerebral ischemic stroke

**DOI:** 10.3389/fncel.2023.1135227

**Published:** 2023-04-05

**Authors:** Guangtao Hu, Cuihong Zhou, Jin Wang, Xinxu Ma, Hongzhe Ma, Huan Yu, Zhengwu Peng, Jing Huang, Min Cai

**Affiliations:** ^1^Department of Psychological Medicine, 958th Hospital, Chongqing, China; ^2^Department of Psychiatry, Xijing Hospital, The Fourth Military Medical University, Xi’an, Shaanxi, China; ^3^Department of Anesthesiology & Perioperative Medicine, Xijing Hospital, The Fourth Military Medical University, Xi’an, Shaanxi, China; ^4^Department of Health Management, Tangdu Hospital, The Fourth Military Medical University, Xi’an, Shaanxi, China

**Keywords:** cerebral ischemia/reperfusion, cannabinoid receptor 1, mitochondrial function, mitochondrial biogenesis, electroacupuncture, depressive-like behaviors, cognitive dysfunction

## Abstract

**Introduction:**

This study aimed to identify the effect of electroacupuncture (EA) treatment on post-stroke depression (PSD) and explore whether cannabinoid receptor 1 (CB1R)-mediated mitochondrial biogenesis accounts for the treatment effect of EA.

**Methods:**

The PSD mouse model was induced by a consecutive 14-day chronic unpredictable stress operation after 7 days of recovery from the bilateral common carotid artery occlusion surgery. Either EA treatment or sham stimulation was performed for 14 consecutive days from Day 7 after the BCCAO operation. Subjects’ PSD-like behaviors were tested via open field test, sucrose preference test, novelty suppressed feeding test, tail suspension test, and forced swim test, and subjects’ cognitive function was examined using Y-maze and novelty object recognition test. In addition, the levels of CB1R, mitochondrial biogenesis-related proteins (nuclear transcription factor 1, NRF1; mitochondrial transcription factor A, TFAM), proteins related to mitochondrial function (Cytochrome C, Cyto C; AIF, COX IV), and mitochondrial DNA were measured. To elucidate the role of CB1R in EA treatment, CB1R antagonists AM251 and CB1R-shRNA were given to mice before EA treatment. Likewise, subjects’ depressive-like behaviors, cognitive function, mitochondrial function, and mitochondrial biogenesis were examined after the PSD procedure.

**Results:**

It has been showed that EA successfully ameliorated depressive-like behaviors, improved cognitive dysfunctions, and upregulated CB1R, NRF1 and TFAM expressions. However, the supplementation of AM251 and CB1R-shRNA blocked the antidepressant-like effects generated by EA, and EA failed to improve cognitive dysfunction, upregulate CB1R protein expression, and increase mitochondrial function and biogenesis.

**Conclusion:**

Altogether, these results indicated that EA ameliorated PSD-like behaviors in mice, improved cognitive dysfunctions after PSD, and promoted mitochondrial biogenesis by activating CB1R, a novel mechanism underlying EA’s antidepressant-like effects in treating PSD.

## Introduction

Stroke, mainly ischemic stroke, is still one of the leading health concerns in China (Wu et al., 2019). At least one-third of stroke survivors suffered from major depression (Post-stroke depression, PSD) and cognitive dysfunction, which leads to increased mortality and mobility of stroke (Jorgensen et al., 2016). However, the specific underlying mechanism of PSD is still under debate, and effective pharmacotherapy against PSD is lacking (Mortensen et al., 2018). The current clinical treatment of PSD is highly dependent on antidepressants, mainly selective serotonin reuptake inhibitors (SSRIs) (Mortensen and Andersen, 2015). Unfortunately, side effects regarding hemorrhage and increased risk of falling limit the use of these medications (Towfighi et al., 2017). Thus, novel medications or strategies for PSD treatment and the exact mechanism underlying PSD urgently need to be explored.

Electroacupuncture (EA), a treatment method derived from traditional Chinese medicine, is one of the most commonly proposed strategies for treating a variety of medical conditions (Wang et al., 2009, 2011). Existing evidence from randomized clinical trials has also indicated that EA treatment elicited antidepressant-like effects but with fewer adverse events (Liu R. et al., 2021; Liu W. et al., 2021). Moreover, some animal studies found that EA alleviated PSD-like behaviors possibly through the mediation of brain-derived neurotrophic factor (BDNF) and via the tyrosine receptor kinase B (TrkB) signaling pathway (Dong et al., 2021; Kang et al., 2021). However, the specific cellular mechanism underlying the treatment effect of EA against PSD remains unclear.

During biochemical processes, mitochondria are the main source of cellular energy and are crucial in maintaining cell function and survival. Impaired mitochondrial function affects numerous neurobiological processes, including a disruption in energy supply to neural cells, excess ROS generation and activation of cytochrome C-regulated mitochondrial apoptotic cascades, and altered synaptic connection, which may exacerbate and progress into central nervous system diseases, including ischemic stroke and mood disorders (Goetzl et al., 2021). Most recent findings supported the relevance between mood disorders and impaired mitochondrial dynamics, including mitochondria morphology and biogenesis. Using post-mortem brain samples, some researchers found that smaller mitochondrial sizecorrelate with lower levels of proteins and ratios between fusion/fission of mitochondria in the brain and peripheral cells (Peric et al., 2020). Similar findings were also detected in rodents with chronic stress, early life stress, and social isolation. With antidepressant treatment, depressive-like behaviors were reversed through the improvement in mitochondrial function (Herbet et al., 2021; Reddy et al., 2021). Together, these findings suggest that mitochondrial biogenesis may play an important role in the pathogenies of PSD and is a potential treatment target of EA, but further research is warranted.

Cannabinoid receptor 1 (CB1R) is a G protein-coupled receptor centrally located in the cellular membrane. In the brain, CB1R is mainly involved in the regulation of neuronal signaling transduction, synaptic plasticity, and so on (Chan and Duncan, 2021). A line of research from our group has demonstrated that CB1R is the crucial mediator of the neuroprotective effect produced by EA (Sun et al., 2016, 2021). Moreover, accumulating evidence from most recent studies, including ours, has demonstrated that cannabidiol and CB1R mediated mitochondrial biogenesis in both neuronal and non-neuronal tissues (Hao et al., 2015). However, to our knowledge, whether EA treatment promotes mitochondrial biogenesis in the experimental PSD model through a CB1R-dependent mechanism is still unknown.

In the current study, we first examined whether EA treatment at the “Baihui (GV20)” acupoint for 14 consecutive days could attenuate depressive-like behaviors and ameliorate cognitive dysfunction in a mouse model of PSD. We then investigated the role of CB1R-mediated mitochondrial biogenesis in antidepressant-like effects and the improvement of mitochondrial function produced by EA pretreatment.

## Materials and methods

### Experimental procedure

In order to assess the role of EA treatment on the PSD-like depressive behaviors and cognitive dysfunction outcome, mice were randomly allocated into three groups (*n* = 8 in each group). The first one was sham group, the second group was PSD group (mice were subjected to BCCAO and subsequently with 2-week CUMS) and the third group was PSD plus EA treatment (mice subjected to BCCAO and then received 2-week CUMS exposure with 2-week EA treatment at the same time). The neurological score was examined at 1, 3 days after BCCAO to screen the mice for the following CUMS. When the operation and treatment were confirmed, the depressive-like behaviors were performed: open-field test (OPT), sucrose preference test, elevated plus maze (EPM), novelty suppressed feeding test (NFST), tail suspension test (TST) and lastly, force swim test (FST). Then the Y-maze test and new object recognition test (NORT) were also performed. After all above tests were confirmed, the mice were decapitated, and hippocampal tissue was collected for the subsequent experiments. The contents of CB1R, proteins related to mitochondrial biogenesis (NRF1, TFAM) and mitochondrial function (Cyto C, AIF and COX IV) were examined by western-blot (*n* = 6 in each group).

To further verify the role of CB1R-mediated mitochondrial function in the anti-depressant and cognitive dysfunction improvement effect produced by PSD, the CB1R small interfering RNA (CB1R-shRNA) and CB1R antagonist AM251 were purchased and used in the current study. Mice were randomly divided into 6 groups: Sham, PSD, EA plus PSD plus Vehicle, EA plus PSD plus AM251, EA plus PSD plus CB1R-shRNA. To determine whether CB1R inhibition or CB1R knockdown affected the antidepressant-like effect and the cognitive dysfunction improvement elicited by the EA treatment, the depressive-like behavior and cognitive outcome were analyzed firstly according to the protocol mentioned above (*n* = 8 in each group). The expression of CB1R, mitochondrial biogenesis and mitochondrial function related proteins, same as above, were also examined by western-blot (*n* = 6 in each group).

### Animals

Male C57Bl6j mice between 8 and 10 weeks old (25–30 g) used in this study were purchased from the Animal Laboratory of the Fourth Military Medical University. All procedures related to animal study were approved by the Ethics Committee for Animal Experimentation of the Fourth Military Medical University (Xi’an, China) and preceded in accordance with the National Institutes of Health Guide for the Care. The mice were maintained in a 12 h alternating light and dark cycle at 20–25°C and 60–70% humidity with water and food freely accessible for at least 1 week before all experiments. A random number table was used for randomization in the present study. The number of animals used and the degree of suffering were minimized.

### Global cerebral ischemic/reperfusion model

The global cerebral ischemic/reperfusion injury was established on the basis of the bilateral common carotid artery occlusion (BCCAO) model. Briefly, mice were anesthetized with 1.5% isoflurane inhalation. Next, a midline incision between the neck and the sternum was made, exposing both common carotid arteries (CCA) located lateral to the sternocleidomastoid muscle. After carefully separating the surrounding tissues and the vagus nerve, CCA was clamped with miniature artery clips to induce global cerebral ischemia. After 15 minutes of ischemia, the clips were removed to restore blood flow. The pericranial temperature was maintained at 37.0 ± 0.5^°^C all through the surgical procedure using a heating pad. Additionally, a laser Doppler flowmeter (PeriFlux System 5000; Perimed, Stock- holm, Sweden) was used to monitor regional cerebral blood flow (rCBF) starting from the anesthetic induction till 5 minutes after reperfusion. Only mice whose rCBF was reduced to < 15% of baseline during ischemia and recovered over 85% during reperfusion stage were included for the following PSD procedure.

To further ensure the success of BCCAO surgery, the neurological measurement was confirmed 1 and 3 days after reperfusion by a trained observer who was blinded to the group assignment. A 10 × 20 cm screen with a 0.2 cm × 0.2 cm grid size that could be rotated from a horizontal to a vertical angle was used in the first test. During the trial, subject was placed on the horizontal screen, and then the screen was rotated into a vertical plane. The duration the mouse could hold onto the screen was recorded for a maximum of 15 s. Next, the mouse was put in the middle of a 1.5 cm-diameter horizontal wooden rod to confirm the second test. The time the mouse could keep to a horizontal rod was recorded for a maximum of 30 seconds. At last, the prehensile traction test was performed to record the duration of the mouse clinging to a horizontal rope for a maximum of 5 s. Each test was worth 3 points, for a total possible 9 points as the final motor score by adding up each test score.

### Post-stroke depression model

The PSD model was established with a consecutive 14-day chronic unpredictable stress operation after 7 days of recovery from the BCCAO surgery in accordance with the methods from our previous report (Tian et al., 2020). Hence, mice were subjected to the stress paradigm once per day. To ensure the stress was unpredictable, different stress stimuli were administrated each day.

### EA treatment

EA treatment strictly followed the procedure described in our previous studies (Sun et al., 2016). The acupuncture needle was located at the acupoint Baihui (GV20). During the treatment, mice were anesthetized with isoflurane and inserted a disposable stainless-steel acupuncture needle to the murine equivalent of the acupoints Baihui. Meanwhile, another electrode was nipped to the tail to the formation of current loop. Then mice were stimulated by EA instrument and maintained with 1.5 MAC isoflurane via nose mask. to restrict its movement. The stimulation was confirmed with an intensity of 1 mA and a frequency of 2/15 Hz for 30 min using the Hwato EA Instrument (Model No. SDZ-V, Suzhou Medical Appliances Co., Ltd., Suzhou, China). The treatment was carried out for a duration of 14 consecutive days, starting from the seventh day after the BCCAO operation.

### Open-field test (OFT)

The OFT was performed in a plastic open field chamber (50 cm length × 50 cm wide × 50 cm depth). A red bulb (25w) located above the center of the chamber was used to dimly illuminate the test room. Mice were placed individually in the same corner of the chamber and allowed to explore the arena feely during the 8-min test duration. An automated video-tracking system (ANY-maze software, Shanghai, China) was applied to record locomotor activity. The apparatus was cleaned with 30% ethanol solution between each session. Total distance traveled and time spent in the center arena during the first 5 minutes were automatically collected for calculation.

### Sucrose preference test (SPT)

The test was operated as described in previous reports with minor modifications (Liu et al., 2018). In brief, mice were single housed with free access to food and trained to adapt to sucrose solution (1%) for 48 h beginning at 7:00 p.m. The positions of water bottle and sucrose bottle were exchanged after the first 24 h. Then the mice were deprived of water and food for 12 hours and underwent the SPT experiment. Tap water and sucrose solution were placed in pre-weighted bottles on the cages, and mice freely consumed the fluids for 12 h in the dark phase. The bottles were weighed again afterward. Sucrose consumption ratio was analyzed by dividing the total amount of sucrose consumed by the total amount of both water and sucrose consumed.

### Elevated plus maze (EPM) test

The EPM apparatus (Mobile Datum, Shanghai, China) consisted of two open arms (30 cm × 5 cm) across from each other, which were perpendicular to two closed arms (30 cm × 5 cm × 15 cm). These arms were connected by a center platform (5 cm × 5 cm). The apparatus was elevated 70 cm above the floor. During each test, a mouse was placed in the central platform facing an open arm and allowed to freely explore the maze for 5 min. The apparatus was cleaned with 30% ethanol solution before and after each test. The location of the mice was tracked and recorded by an automated video-tracking system, and the proportion of entries into open arms and time spent in open arms were calculated.

### Novelty suppressed feeding test (NSFT)

After the EPM test, a novelty-suppressed feeding test was conducted according to procedures described in our previous reports (Blasco-Serra et al., 2017). The test apparatus consisted of a plastic box (50 cm × 50 cm × 40 cm) with a red fluorescent light placed at the center of the arena. The floor was covered wooden bedding, approximately 2 cm thick. Mice were deprived of food but with free access to water for 24 h before the test. During the test, a single pellet of food was placed on the white platform positioned at the center of the box. The behavior of interest (chewing) was scored when the subject bit the food after retrieving the food pellet using forepaws; otherwise, the 6-min test was finished. After the test, mice were immediately put back into their home cage, and the amount of food consumed within those 6 minutes was measured as the food consumption in home cage.

### Tail suspension test (TST)

The tail suspension test was performed following a previously reported method. Each mouse was suspended from a shelf 60 cm above the floor by its tail for 6 minutes. After the first 2 minutes of the test session, the animal’s total amount of immobility time was recorded and calculated. Criteria for immobility and agitation periods were set as being either immobile or agitated for longer than 2 seconds. After 6 minutes of tail suspension, subjects were put back into their home cages.

### Forced swim test (FST)

The forced swim test was performed according to a previous method. Mice were placed individually in a glass testing cylinder (18 cm in height × 30 cm in diameter) containing water 12-cm deep (25 ± 1°C) for 6 minutes. 2 minutes after starting the test, the total duration of immobility (in seconds) was subsequently measured. Immobility was recorded when three paws of the mouse were immobile, and the fourth paw exhibited only minimal movement. After the test session, subjects were gently dried with a paper towel and placed back in their home cages.

### Y-maze test

The function of novelty exploration after PSD was assessed by a three-maze (Y-maze) test. The test apparatus consists of three arms (A, B, and C) (30 cm × 10 cm) positioned at equal angles from each other (120°) and surrounded by various extra-maze cues elevated 45 cm above the floor. During the test, each mouse was placed on one random arm and allowed to explore all three arms freely for 8 min. The entire exploration process and the number and order of the exploration of each arm were automatically tracked and recorded by a video-tracking system. Spontaneous alternation (SA) was identified as the tendency to explore new arms over the one previously chosen. The SA ratio was calculated using the following equation: SA percentage = (number of actual alternations)/(total number of arm entries – 2) × 100.

### Novel object recognition test (NORT)

Cognitive function was also assessed by novel object recognition test. This behavior test consists of three phases in 3 consecutive days: habituation, familiarization, and test phase. During the habituation phase, each mouse was placed into the arena and allowed to freely explore the arena in the absence of objects. Then the mouse was placed back into its home cage. In the familiarization phase, the mouse was placed in the open-filed arena with two identical samples (objects A + A) for 5 min. To avoid coercion to explore the objects, the mouse was released against the center of the opposite wall with its back to the objects. After a 24-hours-retention interval, during the test session, the mouse was placed in the arena with two objects, one identical to the sample and one novel (objects A + B), and allowed to explore the arena freely for 5 min. The training and testing trials were tracked and automatically analyzed by ANY maze software. The relative exploration time was expressed as a discrimination index (D.I. = TN/(TN + TF) × 100%).

### Brain tissue fixation and western blot

Immediately after conducting all the behavioral tests, mice were deeply anesthetized (ketamine, 90 mg/kg and xylazine, 10 mg/kg; *i.p.*) and decapitated. The bilateral hippocampus was harvested, chopped into small pieces, and homogenized in 0.5 ml of RIPA buffer lysis (50 mM Tris–HCl, pH 8.0; 20 mM EDTA; 1% SDS; and 100 mM NaCl). Then the tissue was centrifugated at 12,000 *g* for 15 min, after which the supernatant was collected. For western blot, equal amounts of protein (40 μg) were loaded and probed with primary antibodies as follows: CB1R (Abcam; 1:1000 dilution), NRF-1 (Santa Cruz; 1:500 dilution), TFAM (Santa Cruz; 1:500 dilution), Cytochrome C (Abcam; 1:1000 dilution), AIF (Abcam; 1:1000 dilution), COX IV (Abcam; 1:1000 dilution), and GAPDH (Kangwei; 1:1000 dilution). After washing three times in Tris-buffered saline containing Tween-20, the western-membranes were incubated with secondary horseradish-conjugated goat anti-rabbit secondary antibody at room temperature for 1 hour. The quantitative analysis for all the specific protein bands was made after scanning by an enhanced chemiluminescent reagent (ECL; Millipore). All changes in protein expression were normalized to the ratio of GAPDH.

### Drug and CB1R small interfering RNA administration

The CB1R antagonist, AM251, was dissolved in dimethyl sulfoxide (DMSO) and Tween-80, respectively, followed by dilution in saline. The proportion of DMSO/Tween-80/saline was 1:1:18. Baed on a protocol used in our past study, a dosage of 1 mg/kg for AM251 was injected (*I.P.)* 30 minutes before each EA pretreatment. CB1R-shRNA was purchased from GeneChem Co., Ltd. (Shanghai, China). The target sequences were as follows: sense TGCAGGCCTTCCTACCACTT, antisense TGTGCAGGCAGTCTGAGTCC. Delivery of CB1R-shRNA into the bilateral hippocampus of mice was achieved by micro-injection following the guide cannula. The stereotaxic coordinate location of the hippocampus was (AP: 2.0 mm, ML: 2.5 mm, DV: 2.0 mm).

### Statistical analysis

All statistical analyses were conducted using GraphPad Prism 8.01 software. The neurological scores were expressed as a median with interquartile range and analyzed using the Kruskal-Wallis test, the Mann-Whitney U test, and Bonferroni *post-hoc* test. Other data from depressive-like behavior, cognitive function measurement and protein expression examination were presented as mean ± S.E.M and analyzed using a one-way ANOVA with Bonferroni correction for a *post hoc* correction. A two-tailed *P* < 0.05 was considered statistically significant.

## Results

### EA ameliorated depressive-like behavior in PSD mice

**FIGURE 1:**
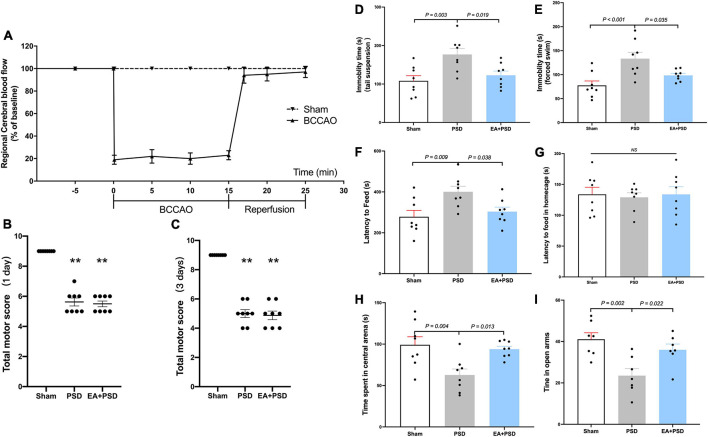
EA treatment alleviated the depressive-like behavior produced by PSD. **(A)** Changes in regional cerebral blood flow (rCBF) during BCCAO operation in mice. **(B,C)** The neuroglial outcome of each group at 1 day and 3 days after reperfusion. (*n* = 8, **P < 0.01 vs. the sham group). **(D)** The treatment of EA reversed the increased immobility in the TST produced by PSD. **(E)** The expose of PSD significantly increased the immobility time in FST, whereas EA treatment reversed this increase. **(F,G)** PSD significantly prolonged the latency to feed during the novel environment in NSF test, while EA treatment prevented this increase. Neither PSD nor EA treatment altered the latency to feed in home cage. **(H)** PSD elicited a significant reduction in the time spent in the central arena of the open field, whereas EA treatment prevented this reduction. **(I)** The expose of PSD significantly reduced time spent in open arms in EPM test, while EA treatment reversed this decrease.

As illustrated in Figure 1A, the results of rCBF changes indicated a successful establishment of BCCAO. Compared with the sham group, total neurological scores were reduced in the PSD and PSD + EA group on day 1 and 3 after reperfusion (Figures 1B, C). No statistical difference was detected between these two groups.

As shown in Figure 1D, in the TST, PSD procedure significantly increased the immobility time (*P* < 0.01), and EA treatment prevented the CUMS-induced increase in immobility time (*P* < 0.05). Figure 1E shows that the immobility time in PSD group was significantly greater as compared with that in the sham group (*P* < 0.001), but this increase was reversed by a 2-week EA treatment (*P* < 0.05). The result of NFST was illustrated in Figure 1F. PSD operation significantly increased the latency to feed in the test field (*P* < 0.01), while EA treatment prevented this increase (*P* < 0.05). In contrast, neither PSD nor EA exerted an effect on the latency to feed in the home cage (*P* > 0.05). As illustrated in Figure 1G, EA treatment prevented the decrease in time spent in the center arena, which was caused by PSD procedure (*P* < 0.05). In EPM test, PSD induced a significant reduction in time spent in open arms (*P* < 0.01), which was significantly reversed by EA treatment (*P* < 0.05).

### EA treatment alleviated the cognitive dysfunction in mice subjected to PSD procedure

**FIGURE 2:**
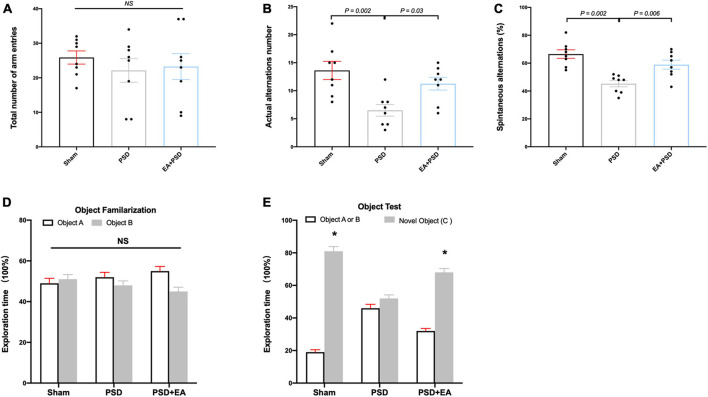
EA treatment improved the cognitive dysfunction which was produced by PSD. **(A–C)** PSD exposure produced a significant impairment of spatial working memory and spatial cognitive ability, which was restored by EA treatment. **(D)** Time of mice spent exploring two identical object A or object B in the phase of familiarization. **(E)** Time of mice spent exploring familiar object A or B and novel object C during the recognition period (*n* = 8, *P < 0.05 vs. familiar object **A** or **B**).

Cognitive function was measured through Y maze test and NORT. In the Y-maze test, significant differences were detected in the SA percentage [Figure 2B, F_(2, 21)_ = 5.926, *P* < 0.01] and the actual alternations number [Figure 2C, F_(2, 21)_ = 4.371, *P* < 0.05] but not in the total number of arm entries [Figure 2A, F_(2, 21)_ = 0.378, *P* = 0.690]. Post hoc test indicated that SA percentage and the number of actual alternations in PSD-exposed group were lower than those in the sham group (*P* < 0.05, respectively), and EA treatment significantly improved spatial cognitive impairment, as indicated by the upregulated alteration behavior in the EA + PSD group.

The exploration time of mice on different objects during object familiarization and novel object recognition phase was examined to calculate the novel object index (Figures 2D, E). As shown in Figure 2D, no significant differences were detected in exploration time between object A and object B among all groups during object familiarization phase (Figure 2D, *P* > 0.05, respectively). During novel object recognition period, the novel object index in the EA + PSD group was significantly greater than that in the PSD group (*P* < 0.01).

### EA treatment increased the expression of CB1R and promoted mitochondrial biogenesis after PSD

**FIGURE 3:**
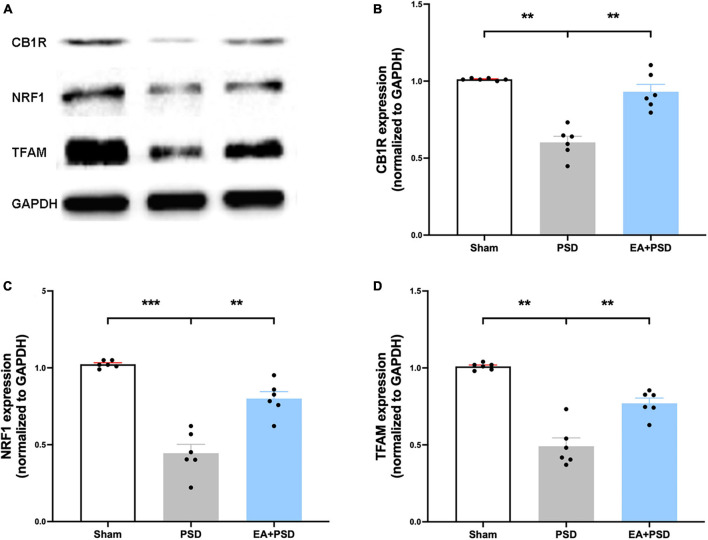
EA treatment improved the CB1R expression and mitochondrial biogenesis. **(A)** Photographs of reprehensive bands of CB1R, NRF1 and TFAM. **(B)** The exposure of PSD inhibited CB1R expression in hippocampus, which was reversed by EA treatment (*n* = 6, **P < 0.01). **(C)** Quantitative changes in NRF1 in the hippocamps after PSD or EA procedure. PSD attenuated the protein expression of NRF1, whereas EA treatment reversed this reduction (*n* = 6, **P < 0.01). **(D)** PSD operation reduced the abundance of TFAM, EA treatment reversed this decrease (*n* = 6, **P < 0.01).

The representative western-blot bands of CB1R, NRF-1, and TFAM were illustrated in Figure 3A. As shown in Figure 3B, the expression of CB1R was reduced in the PSD group as compared with that in the sham group, which was reversed by EA treatment (*P* < 0.05). As demonstrated in Figures 3C, D, in mice treated with EA, the expressions of NRF-1 and TFAM were significantly increased compared to that of the PSD group (*P* < 0.05).

### EA treatment alleviated the mitochondrial dysfunction after PSD

**FIGURE 4:**
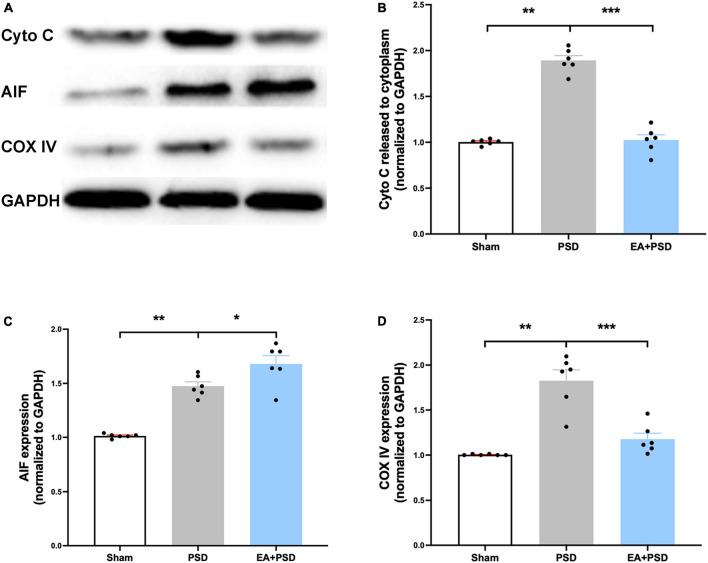
EA treatment ameliorated the mitochondrial dysfunction produced by PSD. **(A)** Representative images of western blot bands of cytochrome C and COX IV protein. **(B–D)** Quantitative changes in cytochrome C **(B)**, AIF **(C)** and COX IV **(D)** in the hippocamps (*n* = 6, **P < 0.01).

All representative western-blot bands related to mitochondrial function (Cyto C, AIF, and COX IV) were presented in Figure 4A. Figure 4B shows that the expression of Cyto C was reduced in the PSD group as compared to the sham group (*P* < 0.01), and was increased in the EA + PSD group (*P* < 0.001). In addition, AIF protein level (Figure 4C) was upregulated in the EA + PSD group (*P* < 0.05). To further determine the effect of EA on mitochondrial function, we examined the protein expression of COXIV, as shown in Figure 4D. Elevated COX IV expression was detected in the mice subjected to PSD (*P* < 0.01). This elevation was significantly reversed by EA treatment (*P* < 0.001).

### The anti-depressive effect of EA was absent in mice administered with CB1R antagonist AM251 or CB1R-shRNA

**FIGURE 5:**
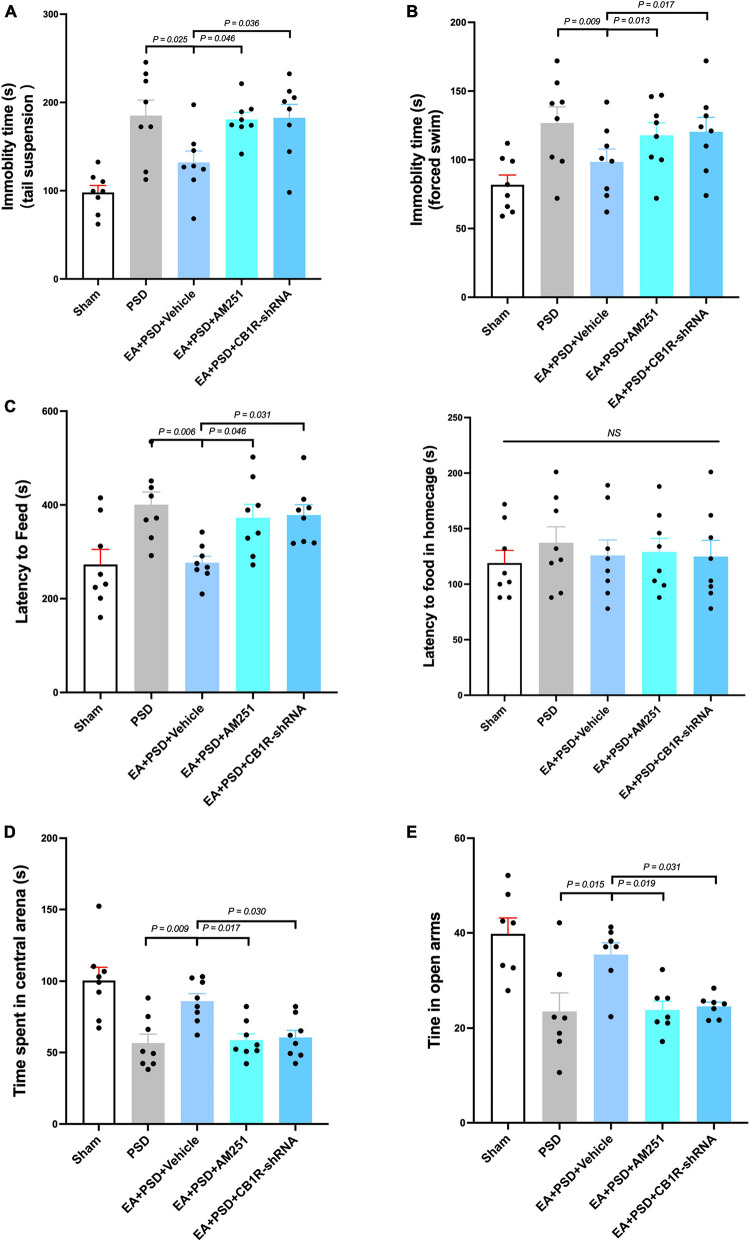
The anti-depressive like behavior produced by EA treatment was abolished by the supplementation of AM251 and CB1R-shRNA. **(A)** Mutant of CB1R by CB1R antagonist AM251 or CB1R-shRNA prevented the decrease in immobility in TST induced by EA treatment. **(B)** CB1R deletion reversed the reduction in immobility in FST produced by EA treatment. **(C)** EA treatment significantly reduced the latency to feed in the novel environment in NSFT, whereas this decreased was reversed by CB1R deficiency. Neither EA nor AM251, CB1R-shRNA altered the latency to feed of mice in their home cages. **(D)** CB1R knockdown significantly abolished the effect of EA treatment on the increase of time spent in the center arena of the OFT. **(E)** In EPM test, EA treatment up-regulated the reduction of time spent in open arm produced by PSD, while CB1R silence prevented this up-regulation (*n* = 8 per group).

The effect of CB1R deficiency on anti-depressive like behaviors produced by EA was illustrated in Figures 5A–E. In TST, AM251 (*P* < 0.05) and CB1R-shRNA (*P* < 0.05), but not vehicle (*P* > 0.05), abolished the decreased immobility time produced by EA (Figure 5A). In Figure 5B, EA treatment significantly reduced the immobility time in FST, which was prevented by either AM251 (*P* < 0.05) or CB1R-shRNA (*P* < 0.05) administration. The result of NFST was presented in Figure 5C. EA significantly decreased PSD-produced reduction in time of latency to feed (*P* < 0.05), but this attenuation was reversed by AM251or CB1R-shRNA administration (*P* < 0.05, respectively). While the vehicle treatment did not alter this reduction related to EA treatment (*P* > 0.05), PSD, AM251, and CB1R-shRNA failed to alter the latency to feed in the home cage (*P* > 0.05). Total distance traveled in OFT was not affected by PSD, EA, AM251, or CB1R-shRNA treatment. The administration of AM251 or CB1R-shRNA reversed the upregulated time spent in the center arena produced by EA in PSD-exposed mice (Figure 5D, *P* < 0.05), whereas the vehicle treatment did not influence that effect. Furthermore, PSD, EA, AM251, or CB1R-shRNA did not alter the time of open-arm entries in EPM. In EA-treated mice, AM251 and CB1R-shRNA reversed the increase in subjects’ time spent in open arms, compared to those in the EA + vehicle group (Figure 5E, *P* < 0.05).

### The improved cognitive function produced by EA was reversed by AM251 and CB1R-shRNA administration

**FIGURE 6:**
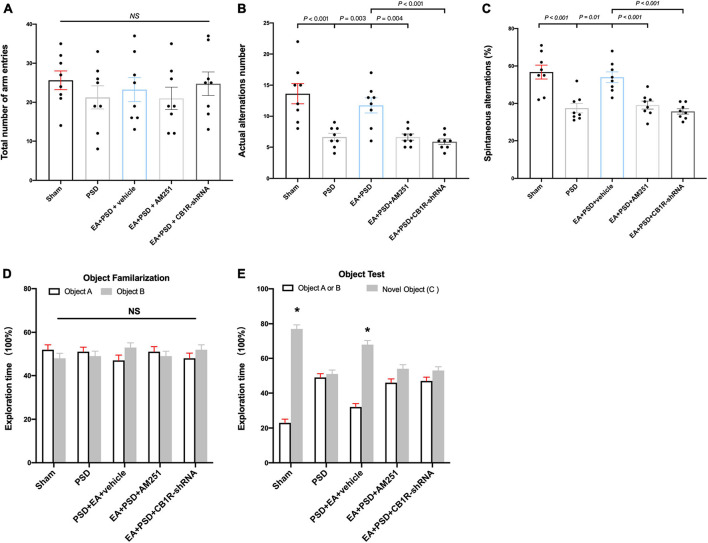
The improved cognitive outcome produced by EA treatment was reversed by the mutant of CB1R. **(A–C)** Neither PSD, EA nor CB1R knockdown affected the number of total arm entries **(A)**. EA treatment significantly improved the exploring outcome of spatial working memory **(B)** and spatial cognitive ability **(C)** in Y-maze test, this effect was reversed by the supplementation of AM251 and CB1R-shRNA. **(D)** The procedure of PSD, EA, AM251 and CB1R-shRNA didn’t alter the time of mice spent exploring two identical object A or object B during the familiarization period. **(E)** EA treatment increased the time of mice spent exploring familiar object A or B and novel object C during the recognition period, whereas this effect was abolished by the deficiency of CB1R (*n* = 8, *P < 0.05 vs. familiar object **A** or **B**).

The role of CB1R deficiency on cognitive function improvement mediated by EA was also explored. In Y-maze test, groups subjected to PSD, EA, AM251, or CB1R-shRNA did not exhibit differences in the total number of arm entries (Figure 6A). EA treatment successfully reversed the decrease in the number of actual alternations and SA percentage produced by PSD procedure; however, these improvements in spatial cognitive function were not evident following the administration of AM251 and CB1R-shRNA (Figures 6B, C).

As shown in Figures 6D, E, no significant differences were detected in object A and object B exploration time among all groups during object familiarization phase (Figure 6D, *P* > 0.05, respectively). in novel object recognition phase, EA treatment increased subjects’ time to explore novel objects; however, this effect was reversed after AM251 and CB1R-shRNA administration. no significant statistical differences were observed between the exploration time spent on familiar and novel objects in the EA + PSD + AM251 group and the EA + PSD + CB1R-shRNA group (Figure 6E, *P* > 0.05, respectively).

### Administration of AM251 and CB1R-shRNA ablated increased CB1R, NRF-1, and TFAM levels produced by EA treatment in PSD mice

**FIGURE 7:**
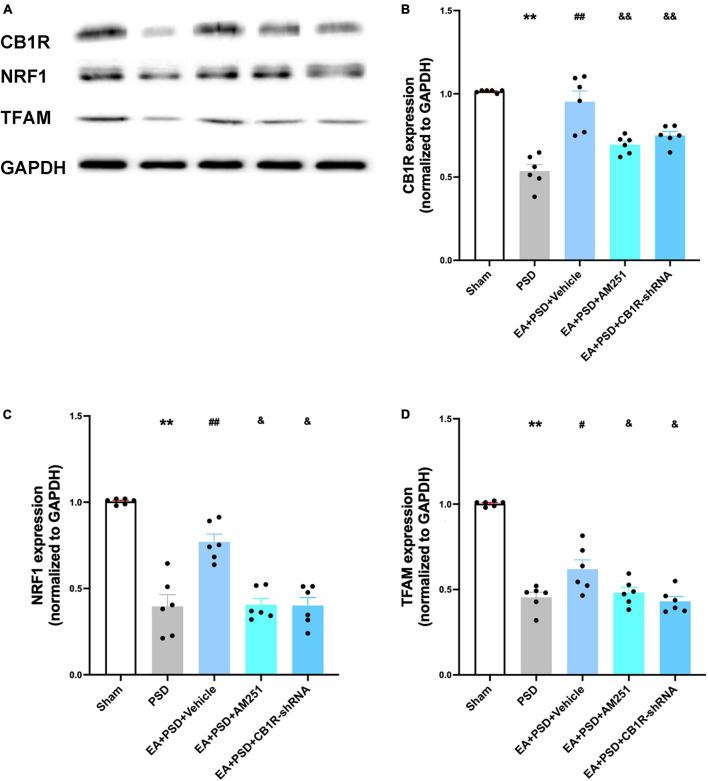
The increased CB1R and mitochondrial biogenesis proteins induced by EA treatment were abolished by AM251 and CB1R-shRNA supplementation. **(A)** Representative images of western blots of the CB1R, NRF-1 and TFAM proteins. **(B)** Quantitative analysis of CB1R protein in hippocamps. **(C,D)** The level of NRF-1 **(C)** and TFAM **(D)** expressed in the hippocampus (*n* =6 per group, *P < 0.05, **P < 0.01 vs. the sham group, ^#^*P* < 0.05, ^##^*P* < 0.01 vs. the PSD group, ^&^*P* < 0.05, ^&&^*P* < 0.01 vs. the EA group).

The representative western-blot bands were presented in Figure 7A. as shown in Figure 7B, In EA animals pretreated with AM251 or CB1R-shRNA, the protein level of CB1R was significantly reduced compared with that of mice in the EA + vehicle group. Moreover, the expression of NRF-1 and TFAM in the EA + AM251 group or EA + CB1R-shRNA group was reduced as compared to that in the EA + vehicle group (Figures 7C, D, *P* < 0.05, respectively).

### Administration of AM251 and CB1R-shRNA reversed the improvement in mitochondrial function produced by EA treatment

**FIGURE 8:**
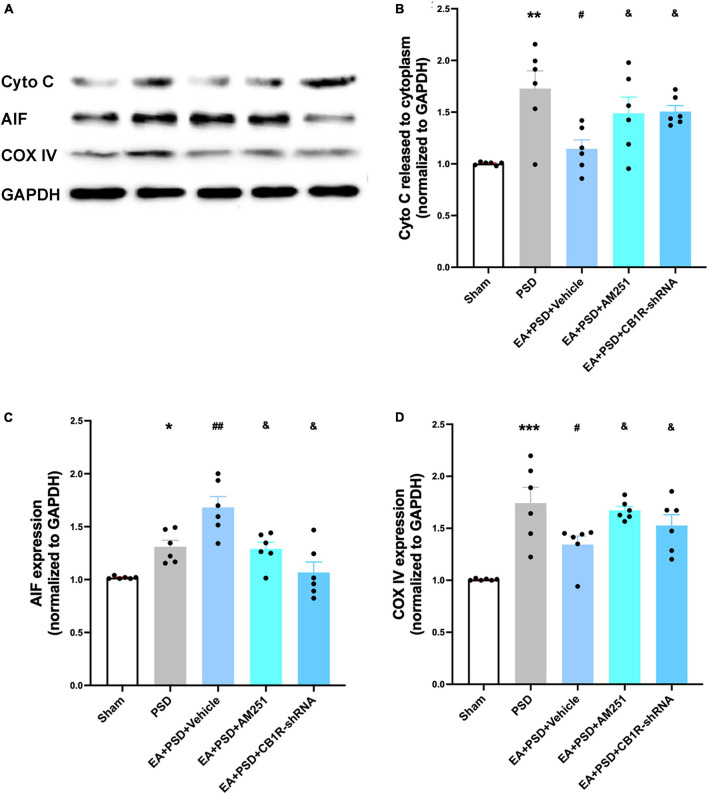
Supplementation with AM251 or CB1R-shRNA reversed the improved mitochondrial function mediated by EA treatment. **(A)** Representative images of western blots of the cytochrome C, AIF and COX ? proteins. **(B,D)** Quantitative changes in cytochrome C **(B)**, AIF **(C)** and COX IV **(D)** and in the in the hippocampus (*n* = 6, *P < 0.05, **P < 0.01, and ***P < 0.01 vs. the sham group, ^#^*P* < 0.05, ^##^*P* < 0.01 vs. the PSD group, ^&^*P* < 0.05 vs. the EA group).

In Figure 8A, the representative western-blot bands in relation to mitochondrial function (Cyto C, AIF, and COX IV) were presented. A reduction in Cyto C protein level expression detected in the EA treatment group was reversed by the administration of AM251 and CB1R-shRNA (Figure 8B, *P* < 0.05). However, the reduction was not statistically significant in the EA + vehicle group. As shown in Figures 8C, D, the expressions of AIF and COX IV in AM251- and CB1R-shRNA-treated mice were significantly lower than those in the EA + vehicle group (*P* < 0.05, respectively).

## Discussion

In the present study, we demonstrated that EA pretreatment was able to improve the depressive-like behaviors and cognitive dysfunction of the PSD mice via CB1R upregulation in the hippocampus. The upregulation of CB1R in the hippocampus significantly promoted mitochondrial biogenesis, which relied on the increased expression of NRF1and TFAM proteins. Also, mitochondrial biogenesis produced by EA pretreatment resulted in the amelioration of mitochondrial function, as evidenced by the increased expression of COX IV and AIF and the reduced expression of Cyto C. The inhibition or silence of CB1R in the hippocampus was able to reverse the effect of EA pretreatment on depressive-like behaviors and cognitive dysfunction. Thus, in line with previous reports, our study provides new insights into exploring the potential treatment maneuvers and understanding the mechanisms of EA in PSD treatment. Moreover, endogenous or exogenous treatment maneuvers aiming to activate CB1R could be a promising target for developing potential therapeutic measures for PSD.

Various experimental models have been developed to mimic PSD-like behaviors in rodents, one of the most applied is the transient global cerebral ischemic model (Flaster et al., 2013; O’Keefe et al., 2014; Vahid-Ansari et al., 2016). Disruption in global blood flow may result in several impaired processes that occur in brain regions, including the hippocampus, striatum, prefrontal cortex, and thalamus (Ahmed et al., 2017; Salaciak et al., 2021). Among these brain regions, the most vulnerable to ischemic/reperfusion insult is the hippocampus, a part of the limbic system that is responsible for emotional responses, mental sensation, and cognitive function (Ressler and Mayberg, 2007; Baranovicova et al., 2022). In line with previous studies, in this research, the injury of hippocampal structure and function exhibited depressive-like behaviors, as evidenced SPT, TST, and NSFT tests. Additionally, the deficiency in hippocampal activity is required for anxiety-like behaviors (Chen et al., 2022; Scholl et al., 2022). Some studies reported by other groups have indicated that anxiety-like behaviors could be generated through ischemic/reperfusion injury (Jin et al., 2017). Consistent with these reports, this study also showed that mice subjected to tGCI/R exhibited more anxiety-related behaviors in OFT and EPM tests. Additionally, hippocampal neuronal damage during ischemia/reperfusion is concomitant with cognitive dysfunction. For instance, some studies noted that tGCI/R impaired spatial learning and memory in Morris water memory test and Barnes maze test (Zhang et al., 2019). In the present study, we utilized Y maze and NOR test, which were also well-accepted behavioral paradigms for testing cognitive function, and found that tGCI/R mice reduced their exploration time in new arms (Y maze test) and toward novel objects (NORT). These results indicated that global ischemia induced long-term spatial memory impairment, consistent with previous publications.

Although some clinical studies have supported the treatment potential of antidepressants, including SSRIs, in treating PSD, antidepressants should be used with caution for PSD patients due to their side effects (Siepmann et al., 2015). In recent years, some meta and systemic reviews demonstrated that EA pretreatment could potentially be an effective and safe monotherapy for PSD (Liu R. et al., 2021; Liu W. et al., 2021). Baihui (GV20, governing vessel meridian 20) is located above the apex auriculate. It has been found that fractional amplitude of low-frequency fluctuation on GV20 could modulate mood-related behaviors (Feng et al., 2019; Xue et al., 2019). A compelling amount of clinical research has shown that the stimulation of GV20 using EA could be an effective monotherapy or adjunctive treatment option for patients with PSD (Chen et al., 2018). Importantly, our previous reports found that EA pretreatment applied at the GV20 point elicited a neuroprotective effect in cerebral ischemic/reperfusion models (Sun et al., 2016, 2021). Moreover, EA at GV20 could effectively alleviate depressive-like and anxiety-like behaviors in MDD and PSD models, improve synaptic plasticity, and inhibit neuro-inflammation response in the hippocampus (Feng et al., 2022; Jia et al., 2022; Li et al., 2022). In accordance with these findings, the current study found that EA intervention at the GV20 point for seven consecutive days after tGCI exerted protective effects against PSD-produced emotional and cognitive dysfunction in mice. However, the mechanisms underlying EA’s antidepressant-like effects are still unclear.

Mitochondria are critically involved in multiple cellular events and biological functions during physical and pathological processes in the CNS. A series of studies have shown that mitochondrial dysfunction plays a vital role in the pathogenesis of depressive-like behaviors (Gimenez-Palomo et al., 2021; Luo et al., 2021). These findings have also implied the rationale behind EA’s potential in ameliorating mitochondrial dysfunction and producing anti-depressant effects (Kasahara et al., 2016; Luo et al., 2021). In CNS, under acute or chronic stress stimuli, the reduction of COX IV and the release of mitochondrial components such as cytochrome C could result in a cascade of damage signals (Yu et al., 2022; Du et al., 2023). Therefore, in this study, we first identified the effect of EA on improving mitochondrial function, as demonstrated by the increased expression of COX IV and AIF and the decreased release of Ctyo C in mitochondria. These results are consistent with our and others’ previous reports using different treatment maneuvers.

To maintain mitochondrial function, mitochondrial biogenesis plays a crucial role in the regulation of mitochondrial quantity and quality (Goldsmith et al., 2022). Accumulating evidence has suggested a strong correlation between mitochondrial biogenesis and mental disorders and identified the promotion of mitochondrial biogenesis as a promising treatment target for conditions like MDD and anxiety (Stachowicz et al., 2016; Goetzl et al., 2021). In the regulation of mitochondrial biogenesis, one of the most studied proteins in signaling pathways are the Nuclear respiratory factor (NRF1) and mitochondrial transcription factor A (TFAM) (Zhu et al., 2020; Shimizu et al., 2022). NRF1 is a vital component in controlling the transcription of nuclear-encoded mitochondrial genes, which affect the electron transport chain and induce the protein expression of TFAM. TFAM initiates mitochondrial DNA replication and mitochondrial-encoded genes transcription. It has been generally accepted that these two proteins expressed in mitochondria are considered indicators of mitochondrial biogenesis due to their major regulatory effect on mitochondrial biogenesis. In particular, our study suggests that the anti-depressive and cognitive promotion effects of EA are related to increased mitochondrial biogenesis regulators NRF1 and TFAM.

EA’s effect in reversing depressive-like behaviors and cognitive dysfunction in tCGI-exposed mice may be associated with the promotion of mitochondrial biogenesis However, no agreeing conclusion has been reached regarding the nature of how EA promotes mitochondrial biogenesis. Therefore, another main question this study seeks to answer is the mechanisms behind this phenomenon. Findings of the current study suggest that EA may promote mitochondrial biogenesis in a CB1R-dependent manner, in agreement with extensive evidence from our group and others’ previous studies (Sun et al., 2016; Hu et al., 2022). Additionally, accumulating evidence has emphasized CB1R and mitochondrial biogenesis’s crucial roles in the PNS and CNS (Bai et al., 2017; Sun et al., 2021). Thus, we applied two different agents, CB1R antagonist AM251 and small interfering RNA, to inhibit CB1R expression in the hippocampus through intraventricular injection. The micro-injection of CB1R-shRNA has been showed as a well-accepted method to knockdown the CB1R expression in brain. Moreover, the current study also applied another wildly-used CB1R-inhibition antagonist, AM251, with the totally different working mechanism to reduce CB1R expression in hippocampus. Consistent with previous experiments, our study revealed that the administration of AM251 or CB1R-siRNA reversed the anti-depressive like behaviors, cognitive promotion effect, and increased mitochondrial biogenesis produced by EA. These results indicate that EA-induced mitochondrial biogenesis occurs as a result of CB1R upregulation.

Although our findings corroborate previous ideas, some limitations still need to be noted in the current study. Firstly, based on our research, we could not conclude that mitochondrial biogenesis was a necessary underlying mechanism of EA’s antidepressant-like effects, for no techniques were applied in this study to directly inhibit mitochondrial biogenesis. Moreover, recent studies have discovered that some signaling pathways, including STAT3 and PGC-1α in mitochondria, mediate the regulation of mitochondrial function. Whether these different regulatory mechanisms participate in this process should be investigated in future experiments.

In summary, this study demonstrated that CB1R-promoted mitochondrial biogenesis is involved in the anti-depressive and cognitive promotion effects of EA. These findings highlight a novel mechanism behind EA’s effects in treating PSD-like behaviors.

## Data availability statement

The original contributions presented in this study are included in the article/supplementary material, further inquiries can be directed to the corresponding authors.

## Ethics statement

The animal study was reviewed and approved by the Ethics Committee for Animal Experimentation of the Fourth Military Medical University.

## Author contributions

GH, JH, and MC designed and conducted the study, collected and analyzed the data. CZ and MC wrote the manuscript. CZ, JW, HM, XM, and ZP participated in the experiment confirm, data collection and analysis. MC and JH supervised the study. All authors approved the final manuscript submission.
